# Development and validation of two nomograms for predicting overall survival and Cancer-specific survival in prostate cancer patients with bone metastases: a population-based study

**DOI:** 10.1186/s12894-023-01372-w

**Published:** 2023-12-05

**Authors:** Baochao Li, Jiajun Xing, Zhongyuan Wang, Zixuan Gong, Zengjun Wang, Aiming Xu

**Affiliations:** Department of Urology, First Affiliated Hospital of Nanjing Medical University, No. 300, Guangzhou Street, Nanjing, 210029 Jiangsu Province China

**Keywords:** Bone metastases, Nomogram, Overall survival, Prostate cancer

## Abstract

**Background:**

Prostate cancer with bone metastasis has significant invasiveness and markedly poorer prognosis. The purpose of this study is to establish two nomograms for predicting the overall survival (OS) and cancer-specific survival (CSS) of prostate cancer patients with bone metastasis.

**Methods:**

From January 2000 to December 2018, a total of 2683 prostate adenocarcinoma with bone metastasis patients were identified from the Surveillance, Epidemiology, and End Results Program (SEER) database. These patients were then divided into a training cohort and a validation cohort, with OS and CSS as the study endpoints. Correlation analyses were employed to assess the relationship between variables. Univariate and multivariate Cox analyses were utilized to ascertain the independent prognostic factors. Calibration curves and the area under the time-dependent receiver operating characteristic curve (time-dependent AUC) were employed to evaluate discrimination and calibration of the nomogram. DCA was applied to examine accuracy and clinical benefits. The clinical utility of the nomogram and the AJCC Stage System was compared using net reclassification improvement (NRI) and integrated discrimination improvement (IDI). Lastly, the risk stratifications of the nomogram and the AJCC Stage System were compared.

**Results:**

There was no collinearity among the variables that were screened. The results of multivariate Cox regression analysis showed that seven variables (age, surgery, brain metastasis, liver metastasis, lung metastasis, Gleason score, marital status) and six variables (age, surgery, lung metastasis, liver metastasis, Gleason score, marital status) were identified to establish the nomogram for OS and CSS, respectively. The calibration curves, time-dependent AUC curves, and DCA revealed that both nomograms had pleasant predictive power. Furthermore, NRI and IDI confirmed that the nomogram outperformed the AJCC Stage System.

**Conclusion:**

Both nomograms had satisfactory accuracy and were validated to assist clinicians in evaluating the prognosis of PABM patients.

## Introduction

Prostate cancer is the most common malignancy of the male reproductive system and the second leading cause of cancer-related mortality worldwide, with adenocarcinoma accounting for 90% of cases [1–3]. Although the use of PSA testing for early detection has improved survival rates, many patients still cannot undergo such testing and are diagnosed in advanced stages [4]. Over 90% of advanced prostate cancer patients develop bone metastasis (BM) [5], shortening median survival time to approximately 1.5–2 years [6]. Prostate cancer often lacks early symptoms, leading to detection in middle and late stages with multiple organ metastases [7]. The bone is the most common site of metastasis, with 10% of new cases diagnosed with BM, increasing to 80% in advanced stages [8]. Patients with BM face severe economic burden, increased mortality risk, and complications such as bone pain, spinal cord compression, and pathological fractures [9].

Bone metastatic prostate cancer leads to a significantly worsened prognosis, and the efficacy of different treatments varies [10], making personalized prediction for patients with prostate adenocarcinoma bone metastasis (PABM) a major focus of research [11]. However, the traditional TNM staging system does not fully reflect the biological behavior of the tumor and the patient’s prognosis, and the staging and grading criteria are relatively isolated, increasing the difficulty for surgeons to evaluate patient prognosis. Moreover, the staging system does not provide clear guidance on treatment strategies for PABM patients.

Nomograms have gained prominence in the field of oncology due to their proven ability to improve predictive accuracy. In recent years, these intuitive and practical models have found widespread application, particularly in the context of personalized medicine, providing a visually intuitive representation of linear prognosis and quantification of individual patient survival [12, 13].In this particular research endeavor, our primary objective is to utilize the extensive dataset available within the Surveillance, Epidemiology, and End Results (SEER) database, specifically focusing on patients with primary atypical meningioma of the brain (PABM), to develop a highly detailed nomogram aimed at predicting the prognosis of PABM patients.

## Method

### Identification of patient population

**Fig. 1:**
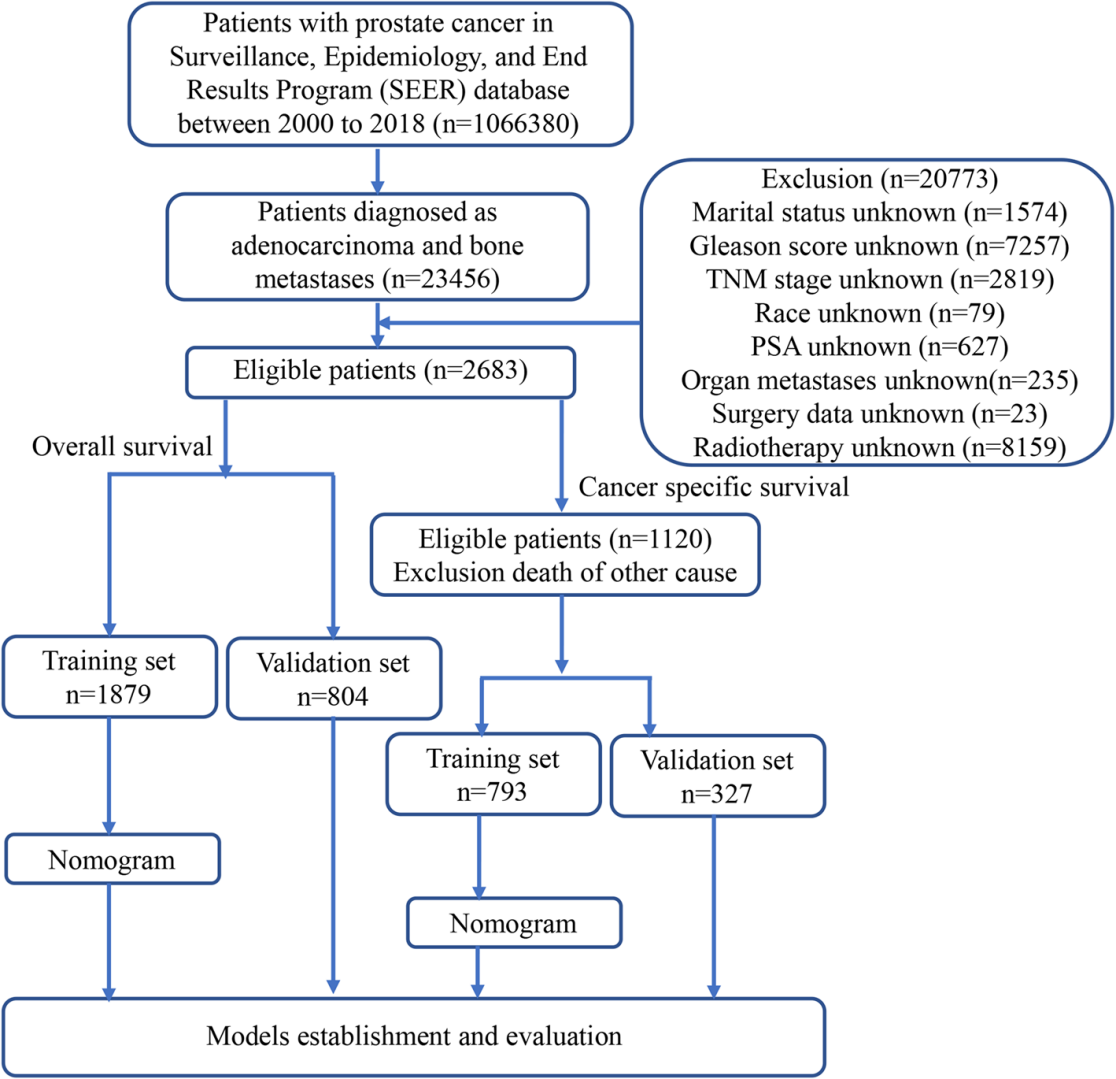
Flow diagram illustrating recruitment of patients

The data for this study were obtained from the Surveillance, Epidemiology, and End Results (SEER) database, which covers approximately 28% of the U.S. population. The SEER database released its data in April 2022, based on cancer incidence in 18 registries across the United States between 2000 and 2018, submitted in Nov 2021. Data abstraction was performed using the SEER*Stat software version 8.4.0. This study included all patients diagnosed with bone metastasis in prostate adenocarcinoma between 2010 and 2018, and the pathologic tumor stage was recorded according to the American Joint Committee on Cancer (AJCC) 7th TNM staging system for prostate cancer. The inclusion criteria: (1) patients diagnosed with prostate adenocarcinoma (PA) (Site recode of ICD-O-3/WHO2008:C61.9 and Histologic Type ICD-O-3:8140); (2) bone metastases (SEER Combined Mets at DX-bone); (3) basic demographic variables, including age, race and gender; (4) complete survival data, follow-up data and specific causes of death; (5) tumor characteristics, including histological information and type, TNM stage; (6) therapeutic measures that whether received surgery, chemotherapy and radiotherapy; (7) known PSA values, Gleason scores at diagnosis; and (8) known metastasis status. The exclusion criteria were as follows: (1) patients were diagnosed only by autopsy or death certificate; (2) patients diagnosed without histological confirmation; (3) patients had more than one primary tumor; (4) patients’ follow-up information was incomplete; (5) patients’ baseline demographic data were unknown or incomplete; (6) patients’ clinicopathological parameter data were unknown or incomplete. Figure 1 provides a detailed illustration of the process for selecting eligible study participants.

### Definition of outcomes

In our study, we wanted to predict 3-year and 5-year overall survival and cancer-specific survival (CSS) in patients with bone metastases from prostate cancer using a column chart that we created using clinical and pathological information. Cancer-specific survival (CSS) is a measure of the likelihood of survival from a specific type of cancer. It is calculated by excluding deaths from other causes and only counting deaths from cancer [14].

### Identification potential covariates

Based on the published literature and the variables included in the SEER database that were applicable to this study, we included in this study lung, liver, and brain metastatic PSA levels and whether or not they were treated with chemotherapy or radiotherapy and surgery, patient age and ethnicity, and marital status, as well as pathological T or N stage and Gleason score. To ensure the reliability and interpretability of the nomogram we aimed to develop, we conducted Spearman’s correlation analysis to examine potential correlations between these selected variables.

### Statistical analysis

We utilized R software (version 4.1.0) for all statistical analyses and developed three models: the Cox-AJCC model, the multifactor Cox model, and the competitive risk model. First, raw data were preprocessed and transformed into factors for subsequent analysis. Pearson correlation analysis assessed potential correlations among variables. The data were then randomly split into training and validation cohorts at a 7:3 ratio. Univariate Cox analysis identified independent variables using *P* values (*P* < 0.1).

To compare significant factors and calculate hazard ratios (HRs) and 95% confidence intervals with a standard of *P* < 0.05, multivariate Cox regression analysis was conducted on variables with differences in univariate Cox regression analysis. Next, a nomogram predicting 3-year, and 5-year overall survival (OS) and cancer-specific survival (CSS) rates were developed using Cox regression based on the training cohort. To evaluate the clinical benefits and utility of the Cox-AJCC model and the multifactor Cox model, and to select the optimal predictive model, the net reclassification index (NRI) and integrated discrimination improvement (IDI) methods were employed. These two complementary validation methods serve different purposes: NRI primarily compares the predictive ability of the old model with the new one by considering improvement at a specific cutoff point, while IDI primarily investigates the overall improvement of the model by examining the model’s overall enhanced performance [15]. For CSS, a competing risk nomogram was created using the Fine-Gray proportional hazards model.

The discriminative performance of the nomograms was assessed through the computation of the area under the curve (AUC) values, which provide a comprehensive evaluation across various thresholds [16]. Furthermore, the predictive accuracy of our nomograms was examined in both the testing and validation cohorts. This evaluation included the assessment of calibration using calibration curves, the utilization of receiver operating characteristic (ROC) analysis, and the application of decision curve analysis (DCA) [17, 18].we also employed Fine-Gray analysis to explore the competing risks associated with our study outcomes, and the cumulative incidence function (CIF) was used to assess the probability of a specific event at a given time point.

## Result

### Demographic characteristics

From the SEER database spanning 2010–2018, we identified 2683 patients suffering from PABM. For OS analysis, they were randomly divided into training (*n* = 1879) and validation groups (*n* = 804). Demographic and clinicopathologic characteristics of all patients are summarized in Table 1, revealing no statistically significant differences between the training and validation cohorts in OS analysis. The majority were under 70 years of age (56.5%), white (77.3%), and married (64.5%). Most had PSA > 20 ng/mL (70.3%) and a Gleason score of 9 (58.0%). Predominant T and N stages were T1 (35.1%), T2 (36.1%), and N0 (68.1%), respectively. The majority of patients had no distant metastases, 97.7% received radiation therapy, and rarely opted for chemotherapy (16.5%) or surgery (11.7%).

**Table 1:** Demographic and clinical characteristics of patients with PABM in OS group

Characteristics	All samples		Training		Validation		*P*
	*N*	Percentage (%)	*N*	Percentage (%)	*N*		Percentage (%)
Age							
< 70	1516	56.50%	1064	56.60%	452	56.20%	0.846
> 70	1167	43.50%	815	43.40%	352	43.80%	
Race							
White	2075	77.30%	1453	77.30%	622	77.40%	0.997
Black	461	17.20%	324	17.20%	137	17%	
Asian/Pacific Islander	130	4.80%	90	4.80%	40	5%	
American Indian/Alaska Native	17	0.60%	12	0.60%	5	0.60%	
Marital status							
Married	1730	64.50%	1216	64.70%	514	63.90%	0.697
other	953	35.50%	663	35.30%	290	36.10%	
Year of diagnosis							
2010–2014	1293	48.20%	895	47.60%	398	49.50%	0.374
2015–2018	1390	51.80%	984	52.40%	406	50.50%	
T stage							
T1	943	35.10%	676	36.00%	267	33.20%	0.295
T2	968	36.10%	682	36.30%	286	35.60%	
T3	447	16.70%	301	16.00%	146	18.20%	
T4	325	12.10%	220	11.70%	105	13.10%	
N stage							
N0	1827	68.10%	1259	67.00%	568	70.60%	0.064
N1	856	31.90%	620	33.00%	236	29.40%	
PSA							
< 4	73	2.70%	52	2.80%	21	2.60%	0.582
4–10	328	12.20%	240	12.80%	88	10.90%	
10–20	395	14.70%	272	14.50%	123	15.30%	
> 20	1887	70.30%	1315	70.00%	572	71.10%	
Gleason score							
< =6	57	2.10%	37	2.00%	20	2.50%	0.9
7 (3 + 4)	163	6.10%	114	6.10%	49	6.10%	
7 (4 + 3)	256	9.50%	178	9.50%	78	9.70%	
8	651	24.30%	452	24.10%	199	24.80%	
9	1556	58.00%	1098	58.40%	458	57.00%	
Chemotherapy							
Yes	443	16.50%	322	17.10%	121	15.00%	0.182
No/Unknown	2240	83.50%	1557	82.90%	683	85.00%	
Radiation							
Yes	2621	97.70%	1836	97.70%	785	97.60%	0.906
No	62	2.30%	43	2.30%	19	2.40%	
Surgery							
Local	248	9.20%	188	10.00%	60	7.50%	0.105
No	2368	88.30%	1646	87.60%	722	89.80%	
Prostatectomy	67	2.50%	45	2.40%	22	2.70%	
lung							
Yes	150	5.60%	103	5.50%	47	5.80%	0.707
No	2533	94.40%	1776	94.50%	757	94.20%	
liver							
Yes	80	3.00%	59	3.10%	21	2.60%	0.461
No	2603	97.00%	1820	96.90%	783	97.40%	
brain							
Yes	27	1.00%	21	1.10%	6	0.70%	0.377
No	2656	99.00%	1858	98.90%	798	99.30%	
Vital status							
Dead	1341	50.00%	949	50.50%	392	48.80%	0.406
Alive	1342	50.00%	930	49.50%	412	51.20%	

**Fig. 2:**
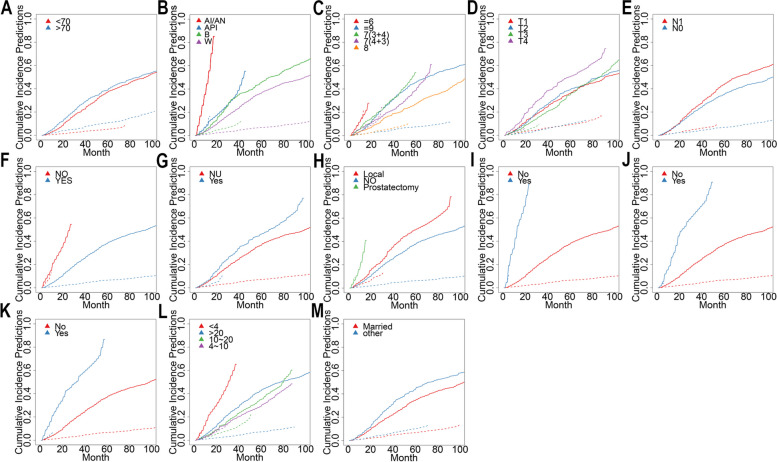
Cumulative incidence predictions of CSS in gastric cancer with liver metastasis. (**A**) Age (**B**) Race (**C**) Gleason Score (**D**) T Stage (**E**) N Stage (**F**) Radiotherapy (**G**) Chemotherapy (**H**) Surgery (**I**) brain (**J**) liver (**K**) lung (**L**) PSA (**M**) Marital Status

The analysis of CSS included 1120 patients, with 793 in the training cohort and 562 in the validation cohort. Although the majority of CSS patients were diagnosed between 2010 and 2014 (70.5%), other demographic characteristics mirrored those in the OS cohort. Table 2 provides a detailed summary of baseline clinical-pathological characteristics. Subgroup analysis of Cumulative Incidence Subgroup analyses of cumulative incidence function (CIF) data showed a higher incidence of CSS in American Indian/Alaska Native patients (Fig. 2B), patients with advanced Gleason scores, and patients with stages T and N (Fig. 2C,D,E). The incidence of CSS was elevated in patients who received chemotherapy (Fig. 2G), lacked radiotherapy (Fig. 2F), and had organ metastases (Fig. 2I, J, K). Surgical intervention significantly increased CIF in PABM patients (Fig. 2H). Interestingly, marriage significantly decreased CIF (Fig. 2M), and both high and low PSA levels were associated with increased CIF (Fig. 2L). Patients over 70 years of age also exhibited higher CIF, but the results were not significant (Fig. 2A).

**Table 2:** Demographic and clinical characteristics of patients with PABM in CSS group

Characteristics	All samples		Training		Validation		*P*
	*N*	Percentage (%)	*N*	Percentage (%)	*N*	Percentage (%)	
Age							
< 70	640	57.10%	459	57.90%	181	55.40%	0.437
> 70	480	42.90%	334	42.10%	146	44.60%	
Race							
White	856	76.40%	593	74.80%	263	80.40%	0.240
Black	220	19.60%	166	20.90%	54	16.50%	
Asian/Pacific Islander	34	3%	26	3.30%	8	2.40%	
American Indian/Alaska Native	10	0.90%	8	1%	2	0.60%	
Marital status							
Married	690	61.60%	477	60.20%	213	65.10%	0.119
other	430	38.40%	316	39.80%	114	34.90%	
Year of diagnosis							
2010–2014	790	70.50%	545	68.70%	245	74.90%	0.039
2015–2018	330	29.50%	248	31.30%	82	25.10%	
T stage							
T1	394	35.20%	283	35.70%	111	33.90%	0.551
T2	413	36.90%	297	37.50%	116	35.50%	
T3	153	13.70%	107	13.50%	46	14.10%	
T4	160	14.30%	106	13.40%	54	16.50%	
N stage							
N0	742	66.20%	516	65.10%	226	69.10%	0.193
N1	378	33.80%	277	34.90%	101	30.90%	
PSA							
< 4	32	2.90%	22	2.80%	10	3.10%	0.381
4–10	85	7.60%	67	8.40%	18	5.50%	
10–20	134	12%	92	11.60%	42	12.80%	
> 20	869	77.65%	612	77.20%	257	78.60%	
Gleason score							
< =6	12	1.10%	8	1%	4	1.20%	0.625
7 (3 + 4)	58	5.20%	43	5.40%	15	4.60%	
7 (4 + 3)	78	7%	59	7.40%	19	5.80%	
8	240	21.40%	162	20.40%	78	23.90%	
9	732	65.40%	521	65.70%	211	64.50%	
Chemotherapy							
Yes	198	17.70%	145	18.30%	53	16.20%	0.407
No/Unknown	922	82.30%	648	81.70%	274	83.80%	
Radiation							
Yes	1099	98.10%	778	98.10%	321	98.20%	0.949
No	21	1.90%	15	1.90%	6	1.80%	
Surgery							
Local	119	10.60%	90	11.30%	29	8.90%	0.264
No	993	88.70%	696	87.80%	297	90.80%	
Prostatectomy	8	0.70%	7	0.90%	1	0.30%	
lung							
Yes	85	7.60%	59	7.40%	26	8%	0.769
No	1035	92.40%	734	92.60%	301	92%	
liver							
Yes	58	5.20%	41	5.20%	17	5.20%	0.984
No	1062	94.80%	752	94.80%	310	94.80%	
brain							
Yes	19	1.70%	16	2%	3	0.90%	0.195
No	1101	98.30%	777	98%	324	99.10%

### Correlations among variables

**Fig. 3:**
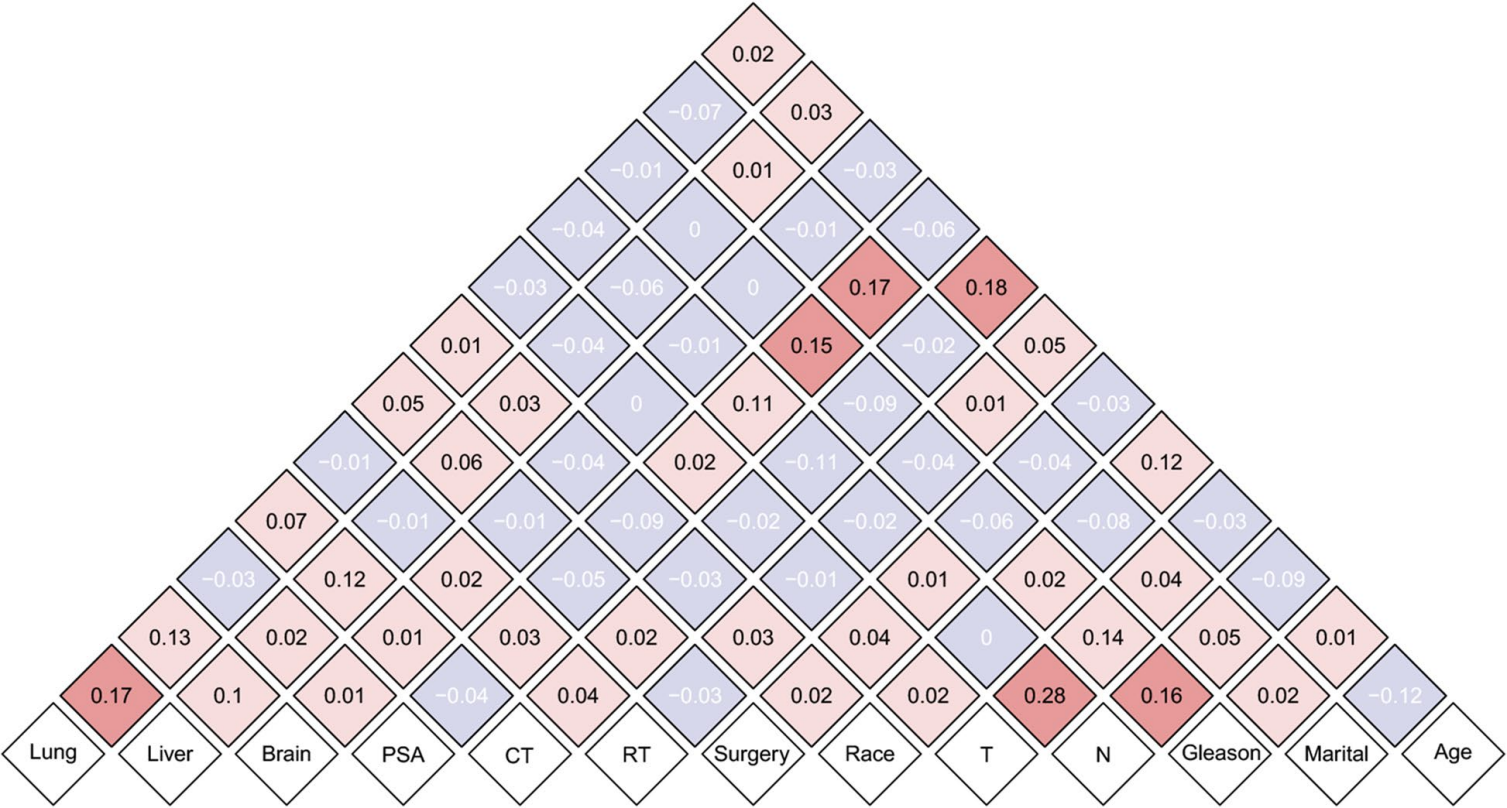
The results of correlation analysis between all included variables. CT: chemotherapy; RT: radiotherapy

Before performing Cox regression analysis, we first checked for covariance between the variables of interest using Spearman’s correlation analysis. The results are shown in Fig. 3. We found that T staging and N staging had the strongest correlation of all variables, with a correlation coefficient of 0.28. We also observed a relatively significant positive correlation between chemotherapy and age, with a correlation coefficient of 0.18. In addition, both lung and liver metastases showed a correlation coefficient of 0.17 with PSA levels and marital status, respectively. This is closely followed by Gleason score and N staging and PSA levels, with correlation coefficients of 0.16 and 0.15 respectively. Of note, there was a mild negative association between age and marital status, with a correlation coefficient of − 0.12.Nevertheless, most of the variables showed relatively low correlations, with values ranging from − 0.1 to 0.1, suggesting that there was no significant correlation for the included variables.

### Evaluation of nomogram variables

Cox regression analysis revealed a model including factors such as age, surgical intervention, brain, liver and lung metastases, Gleason score and marital status that had the most significant *P* value in the training set. In total, seven variables showed a significant correlation with overall survival (OS) in univariate analysis. Further multivariate Cox regression analysis revealed that factors such as age ≥ 70 years (HR = 1.404, 95% CI = 1.233–1.598, *P* < 0.001), prostatectomy (HR = 0.356, 95% CI = 0.180–0.703, *P* = 0. 003), having a Gleason score ≥ 9 (HR = 2.774, 95% CI = 1.629–4.723, *P* < 0.001) and being married (HR = 1.390, 95% CI = 1.218–1.587, *P* < 0.001) emerged as independent prognostic factors for PABM patients. Table 3 provides a more complete breakdown of these details.

**Table 3:** Univariate and multivariate Cox proportional hazards regression analysis of patients with PABM in the OS group

Variables	Univariate Cox regression analysis			Multivariate Cox regression analysis		
	HR	95% CI	*P*	HR	95% CI	*P*
age						
<70	1 (reference)				1 (reference)	
≥ 70	1.499	1.313–1.712	0.000	1.404	1.233–1.598	0.000
Race						
American Indian/Alaska Native	1 (reference)					
Asian/Pacific Islander	0.667	0.304–1.466	0.313			
Black	0.993	0.481–2.052	0.985			
White	0.802	0.392–1.639	0.545			
T Stage						
T1	1 (reference)					
T2	0.956	0.822–1.112	0.560			
T3	0.829	0.670–1.026	0.084			
T4	1.063	0.855–1.323	0.582			
N Stage						
N1	1 (reference)					
N0	0.922	0.798–1.065	0.268			
Surgery						
local	1 (reference)			1 (reference)		
NO	0.835	0.681–1.024	0.084	0.831	0.680–1.015	0.070
Prostatectomy	0.468	0.232–0.944	0.034	0.356	0.180–0.703	0.003
Brain Metastasis						
No		1 (reference)			1 (reference)	
Yes	2.227	1.292–3.840	0.004	2.135	1.250–3.646	0.000
Liver Metastasis						
NO	1 (reference)			1 (reference)		
Yes	2.234	1.588–3.145	0.000	2.292	1.642–3.200	0.000
Lung Metastasis						
No	1 (reference)			1 (reference)		
Yes	1.337	1.010–1.770	0.042	1.438	1.088–1.902	0.011
Gleason score						
≤ 6	1 (reference)			1 (reference)		
7(3 + 4)	1.605	0.888–2.900	0.117	1.718	0.955–3.092	0.071
7(4 + 3)	1.589	0.892–2.834	0.116	1.815	1.025–3.214	0.041
8	1.391	0.800–2.420	0.242	1.622	0.942–2.794	0.081
≥ 9	2.359	1.370–4.061	0.002	2.774	1.629–4.723	0.000
PSA						
<4	1 (reference)					
4 ~ 10	0.659	0.420–1.032	0.069			
10 ~ 20	0.812	0.526–1.252	0.345			
>20	1.033	0.690–1.547	0.874			
Marital						
Married	1 (reference)			1 (reference)		
other	1.284	1.120–1.472	0.000	1.390	1.218–1.587	0.000

Regarding the CSS-based classification, the specifics of the CSS group members diagnosed with PABM are described in Table 4. CSS-related prognostic factors for patients diagnosed with PABM were identified by univariate Cox regression analysis, adjusting for age, surgical procedures, liver or lung metastases, Gleason score and marital status. A subsequent multivariate Cox regression analysis was performed to identify the independent prognostic variables for PABM patients. These observations are shown in Table 4.

**Table 4:** Results of univariate and multivariate analyses by Fine-Gray proportional subdistribution hazards model

Variables	Univariate Cox regression analysis			Multivariate Cox regression analysis		
	HR	95% CI	*P*	HR	95% CI	*P*
age						
<70	1 (reference)				1 (reference)	
≥ 70	1.277	1.098–1.485	0.001	1.162	1.003–1.346	0.045
Race						
American Indian/Alaska Native	1 (reference)					
Asian/Pacific Islander	0.524	0.228–1.205	0.128			
Black	0.868	0.408–1.849	0.714			
White	0.677	0.321–1.425	0.304			
T Stage						
T1	1 (reference)					
T2	0.989	0.832–1.175	0.897			
T3	0.903	0.717–1.137	0.384			
T4	1.186	0.924–1.521	0.181			
N Stage						
N1	1 (reference)					
N0	0.906	0.770–1.066				
Surgery						
local	1 (reference)			1 (reference)		
NO	0.869	0.691–1.095	0.234	0.873	0.698–1.091	0.231
Prostatectomy	0.439	0.203–0.952	0.037	0.341	0.162–0.719	0.005
Brain Metastasis						
No		1 (reference)				
Yes	1.840	0.652–5.189	0.249			
Liver Metastasis						
local	1 (reference)			1 (reference)		
NO	2.522	1.644–3.870	0.000	2.8673	1.906–4.313	0.000
Lung Metastasis						
local	1 (reference)			1 (reference)		
NO	1.441	1.022–2.031	0.037	1.714	1.250–2.351	0.001
Gleason score						
≤ 6	1 (reference)			1 (reference)		
7(3 + 4)	2.048	0.956–4.388	0.065	2.271	1.055–4.887	0.036
7(4 + 3)	2.090	1.002–4.361	0.049	2.458	1.168–5.169	0.018
8	1.929	0.945–3.940	0.071	2.329	1.137–4.770	0.021
≥ 9	3.382	1.668–6.856	0.001	4.194	2.068–8.507	0.000
PSA						
<4	1 (reference)					
4 ~ 10	0.626	0.392–1.000	0.050			
10 ~ 20	0.714	0.456–1.118	0.141			
>20	0.994	0.657–1.505	0.977			
Marital						
Married	1 (reference)			1 (reference)		
other	1.228	1.052–1.433	0.009	1.325	1.144–1.535	0.000
Chemotherapy						
No		1 (reference)				
Yes	1.100	0.906–1.336	0.334			

### Nomogram development and validation

**Fig. 4:**
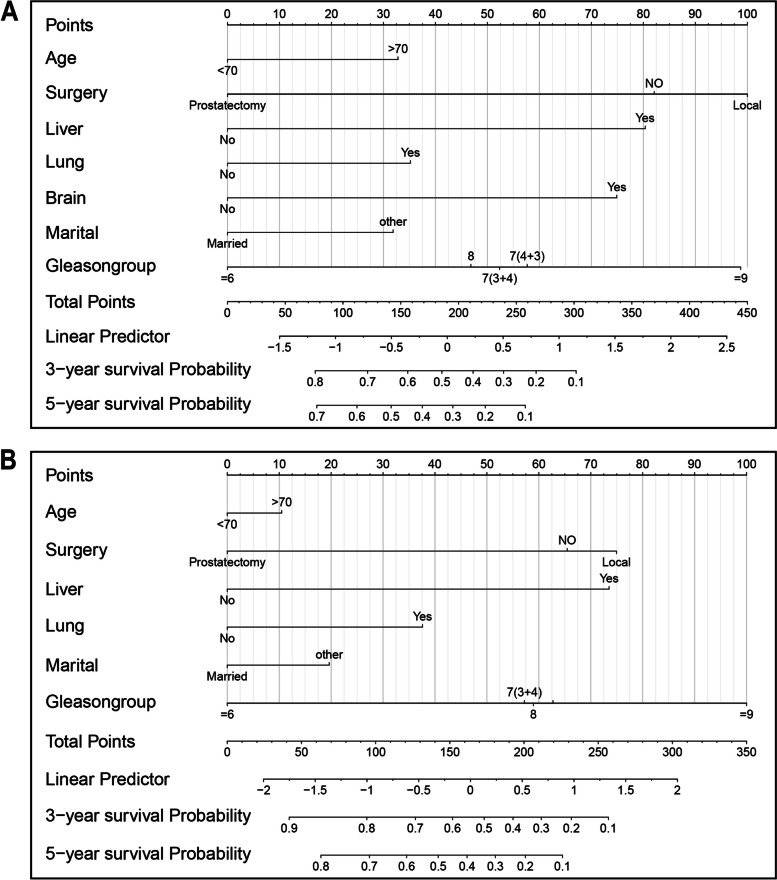
Constructed nomograms for prognostic prediction of overall survival and cancer specific survival

**Fig. 5:**
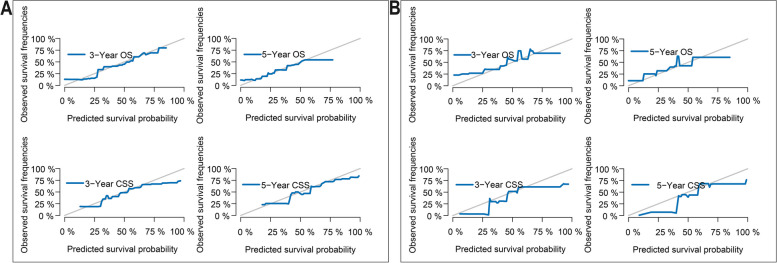
Calibration curves. **A** 3-year and 5-year likelihoods of OS and CSS in the training dataset. **B** 3-year and 5-year likelihoods of OS and CSS in the validation dataset

To predict 3-year and 5-year CSS and OS for patients diagnosed with PA and bone metastases, we formulated two different nomograms using independent prognostic determinants derived from multivariate Cox and CSS analyses (Fig. 4A,B). These well-established nomograms can be used to assess the potential influence of different factors that either augment or directly induce mortality in PA patients by aggregating the individual variables. The calibration curves of our nomograms showed remarkable agreement between expected and actual outcomes in both training and validation subsets (Fig. 5).

**Fig. 6:**
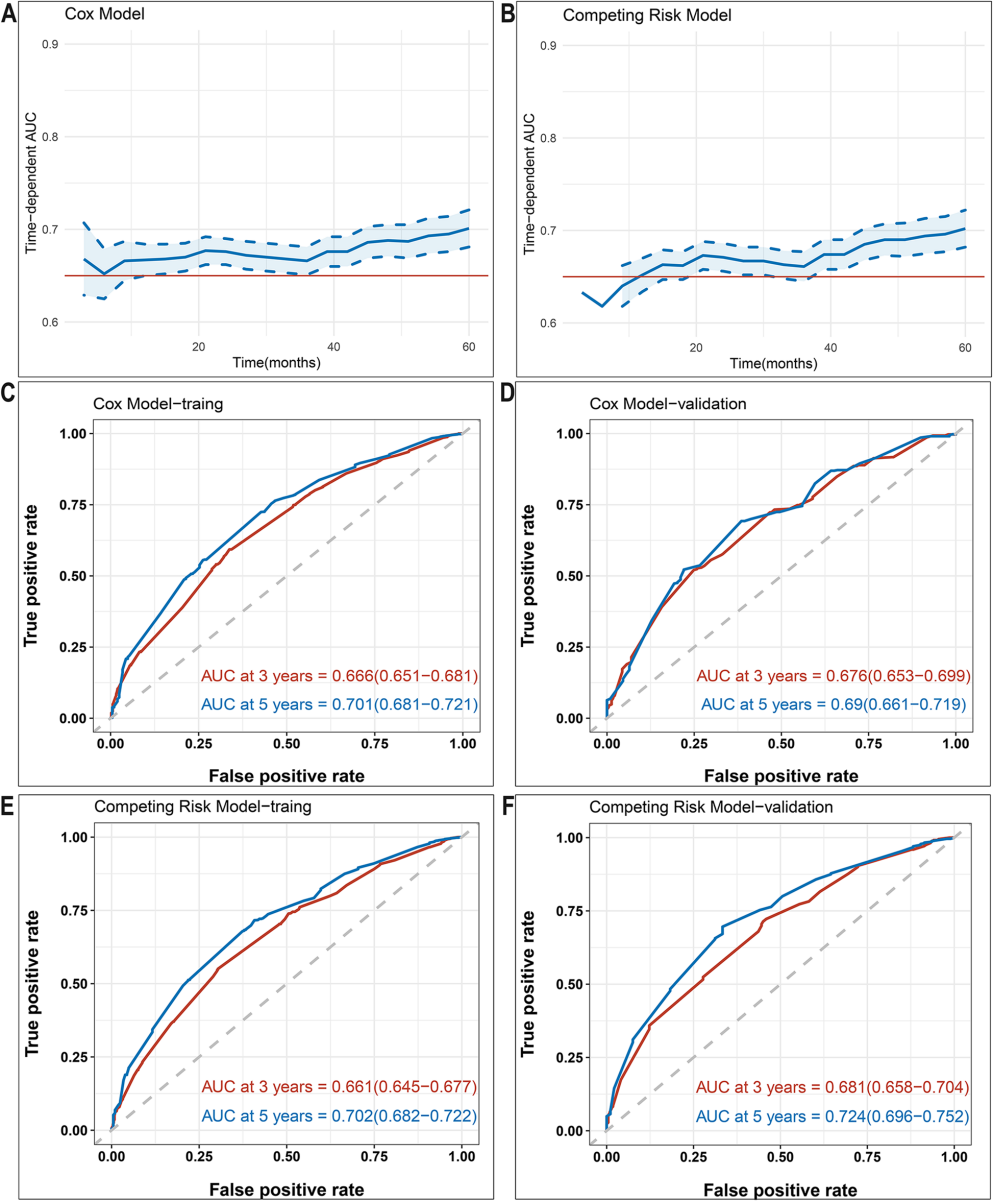
Time-dependent AUC and receiver operating characteristic (ROC) curves of OS and CSS. **A**,**B** Time-dependent AUC of using the nomogram to OS and CSS probability within 5 years in the training cohort and validation cohort. The blue line represents AUC = 0.65. And the shading area between blue dotted curves represents 95% credible intervals. **C**,**D** ROC curves corresponding to 3-year and 5-year OS in the training and validation cohort, respectively. **E**,**F** ROC curves corresponding to 3-year and 5-year CSS in the training and validation cohort, respectively

The AUC curves for the Cox and Competing risk models by time period were presented in Fig. 6A and B, respectively. Based on our analysis, the nomogram demonstrated good discriminatory power in predicting OS and CSS within 5 years, as shown by AUC values greater than 0.65. Specifically, in the training cohort, the Cox model exhibited AUC values of 0.666 and 0.701 at 3 and 5 years, respectively (Fig. 6C). The corresponding AUC values in the validation cohort were 0.676 and 0.690 (Fig. 6D). Similarly, the competing risk model showed AUC values of 0.661 and 0.702 at 3 and 5 years, respectively, and the corresponding values in the validation group were 0.681 and 0.724 (Fig. 6E,F).

**Fig. 7:**
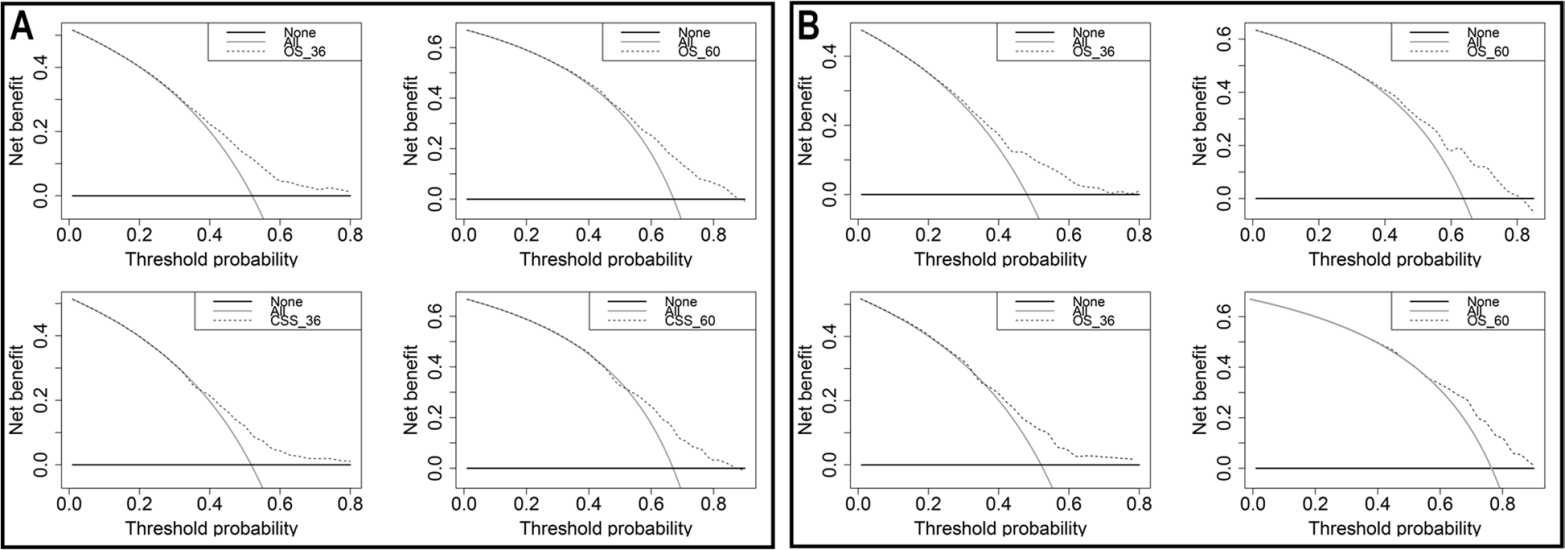
Decision curve analysis of the nomogram in the estimation of OS and CSS of patients with PABM. **A** Training cohort. **B** Validation cohort. The None line illustrates the net benefit when assuming no patients die; the All line represents the net benefit if all patients die

Decision curve analyses were performed on both the training and validation samples and the results are shown in Fig. 7. The decision curves for 3-year and 5-year survival showed optimal net benefits, highlighting the improved predictive accuracy of the established nomogram.

### Comparison of clinical performance between the nomograms and the AJCC staging system

To further explore the clinical applicability of the nomograms proposed here, we used the NRI, IDI and C-index metrics for a comparative assessment of the precision of the nomogram against the AJCC staging methodology. Within the training subgroup, our nomogram registered a C-index of 0.6333 (95% CI = 0.5888–0.6390) in contrast to the AJCC system, which registered 0.5413 (95% CI = 0.5167–0.5676). Additionally, the NRI values for 3-year and 5-year OS were 0.3697 (95% CI = 0.2597–0.5146) and 0.4465 (95% CI = 0.2952–0.5801), respectively. Meanwhile, the IDI values for 3-year and 5-year OS were 0.05 (95% CI = 0.030–0.074, *P* < 0.001) and 0.053 (95% CI = 0.030–0.080, *P* < 0.001), respectively (as shown in Table 5). Analogous results were observed in the validation subset (as shown in Table 5), reinforcing the notion that our nomogram has improved prognostic ability when compared to the AJCC staging system.

**Table 5:** Comparison of different models for estimating the overall survival of PABM patients

	Training cohort			Validation cohort		
Index	Estimate	95%CI	*P* value	Estimate	95%CI	*P* value
NRI (vs. AJCC stage System)						
For 1-year OS	0.428	0.238–0.557		0.433	0.137–0.645	
For 3-year OS	0.370	0.260–0.515		0.403	0.136–0.570	
For 5-year OS	0.446	0.295–0.580		0.533	0.199–0.737	
IDI (vs. AJCC stage System)						
For 1-year OS	0.044	0.025–0.071	< 0.0001	0.050	0.024–0.091	< 0.0001
For 3-year OS	0.050	0.030–0.074	< 0.0001	0.041	0.005–0.084	0.028
For 5-year OS	0.053	0.030–0.080	< 0.0001	0.073	0.001–0.165	0.044
C-Index						
The nomogram (OS)	0.633	0.589–0.639		0.639	0.559–0.637	
AJCC Stage System	0.541	0.517–0.568		0.542	0.491–0.569	

### Risk classification for prostate cancer patients with bone metastases

**Fig. 8:**
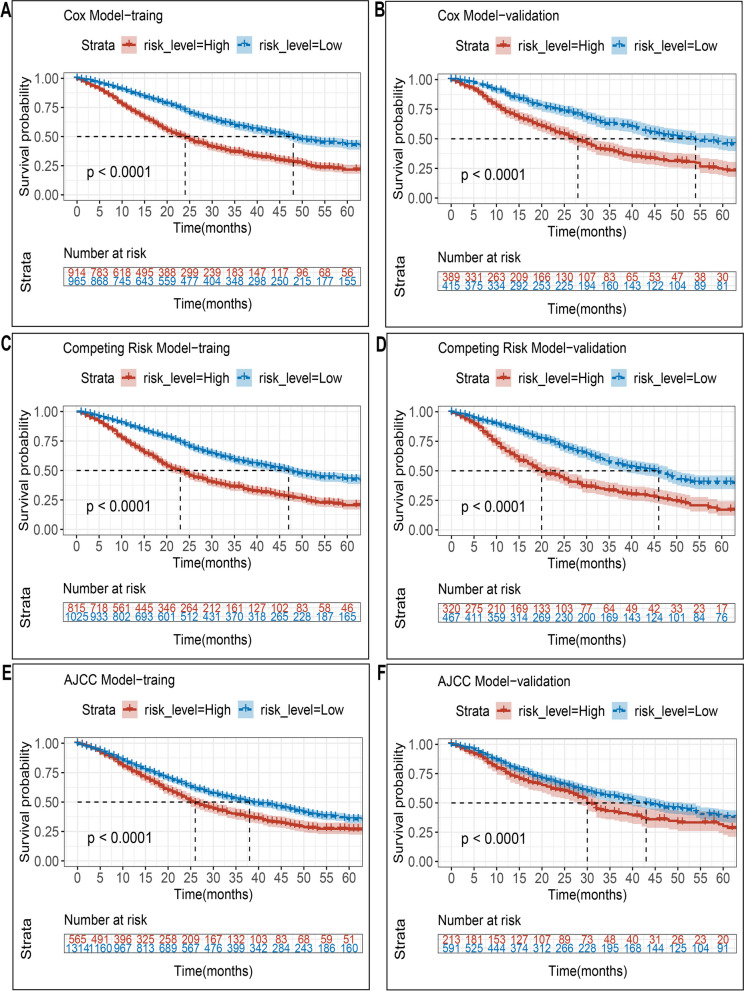
Kaplan–Meier OS and CSS curves of PABM patients with different risks stratified by the nomogram. **A**,**B** PABM patients in the training and validation cohort at different stages are classified according to the AJCC staging system. **C**,**D** PABM patients in the training and validation cohort at different stages are classified according to the cox model nomogram. **E**,**F** PABM patients in the training and validation cohort at different stages are classified according to the competing risk model nomogram

We stratified the risk based on the total scores calculated from the nomogram, which enabled the classification of each PA patient into two risk groups (low-risk and high-risk) based on the median value. Kaplan-Meier survival analysis revealed that the low-risk group had significantly better OS and CSS than the high-risk group (Fig. 8C–F). Furthermore, the nomogram exhibited excellent discriminatory power between the two risk groups, while the AJCC Stage System had limited capability to distinguish between them (Fig. 8A,B). These findings were replicated in the validation cohort.

## Discussion

Prostate cancer is one of the most common cancers in men, and its incidence is increasing every year [19]. Bones are the most common site of distant metastasis, and elderly men are often diagnosed with advanced prostate cancer due to bone metastases, which often indicate a poor prognosis [20, 21]. Adenocarcinoma is the most common pathological type of prostate cancer, accounting for over 90%, therefore, in this study, we chose adenocarcinoma as the subject of our research. Early detection and limited treatment options for PABM require urgent solutions. In our study, we constructed two predictive models to predict the prognosis of PABM patients. The validation of our nomograms revealed that they exhibit strong discriminative performance and calibration. Furthermore, our risk stratification approach successfully categorized PABM patients into high- and low-risk groups, demonstrating a significant difference between the two groups.

It has been reported that several factors affect the survival of patients with prostate adenocarcinoma bone metastasis (PABM), including age, Gleason score, PSA level, clinical stage, and treatment modality [22, 23]. Therefore, we attempted to incorporate as much information as possible from the SEER database into Cox and competing risk models to predict the prognosis of PABM patients. According to Zhang, Z. et al. study [24], age significantly affects the prognosis of bone-metastatic prostate cancer patients. In our study, we also found that advanced age was closely associated with a poorer prognosis in PABM patients, as demonstrated by both the CSS and OS models. The Gleason score is an important indicator for evaluating the degree of differentiation of prostate cancer. A higher score often indicates a lower degree of tumor differentiation, stronger invasiveness, and a poorer prognosis [22, 25]. In addition to the Gleason score, PSA value and clinical T/N stage are generally considered strong prognostic factors for PCa and are often combined to assess the risk of death in PCa patients with bone metastasis [26]. Our predictive model achieved results that are generally consistent with previous studies. Furthermore, with regard to PABM patients should undergo surgery, and what type of surgery to choose, has always been a controversial topic in metastatic prostate cancer. We have found that for PABM patients, aggressive surgical treatment can actually shorten their survival time. However, in the study by Qin XJ et al. [27], tumor cytoreduction surgery such as TURP can reduce PSA levels during follow-up, reduce tumor burden, alleviate urinary obstruction and pain, thus relieving disease-related suffering. However, surgical complications may worsen the prognosis, so the patient’s overall condition should be thoroughly evaluated before surgery to truly improve the patient’s condition [28].

Chemotherapy has been proven to be important in the treatment of bone metastatic prostate cancer, according to several clinical trials [29, 30]. Docetaxel is one of the most commonly used chemotherapeutic drugs for bone metastatic prostate cancer, which can prolong the survival of patients [29]. However, our competing risk model showed that chemotherapy increased the risk of death in patients with prostate cancer and bone metastasis. This may be closely related to the incomplete information about chemotherapy in the SEER database. In fact, if patients are fit enough, chemotherapy is recommended along with castration in newly diagnosed M1 phase [30].

Radiotherapy is also an important treatment for prostate cancer and plays an important role in metastatic disease. In our study, radiotherapy significantly reduced the Cumulative incidence predictions of CSS in PABM patients, thereby prolonging the tumor-specific survival time. Similarly, the SEER database’s limitations in documenting radiotherapy information may affect the precision of our conclusions. Parker CC’s three-phase clinical study confirmed that radiotherapy can significantly improve the survival rate of low metastatic load PC patients, but the radiation dose still needs further research to clarify and formulate standardized radiotherapy guidelines [31].

In addition to the aforementioned factors, marital status has been recognized as potential predictors of survival in prostate cancer patients [32, 33]. The effect of marital status on survival has also been previously reported [34]. Our findings are consistent with this, indicating that married patients tend to have a better survival advantage than single patients [35], likely due to the provision of explicit treatment and emotional support [36]. This was consistent with our study. Consistent with prior research findings [37], our study reveals that unmarried PCa patients are more likely to develop progressive disease. This increased risk could potentially be linked to changes in male hormones following marriage, as it is widely recognized that androgens play a critical role in the development of PCa [38].

Besides bone metastasis, we also investigated liver, brain, and lung metastasis, even though patients diagnosed with these distant sites accounted for only a small portion of the SEER database. In this study, we found that lung metastasis (5.59%) was the most common distant metastatic site, followed by liver (2.98%) and brain (1.01%), and the probability of combined metastases was consistent with previous reports. When distant metastasis was detected, PCa cells had already spread to multiple organs, which is consistent with the results of several previous studies [39, 40]. This also indicates that patients with liver, brain, or lung metastases were more likely to have bone metastasis, and multiple site metastases together influenced the prognosis of PABM patients. In our CSS model, lung, liver, and brain metastases significantly increased the CIF value, resulting in an increased risk of competing death events for PABM patients.

Despite the valuable insights provided, our study has some limitations. Firstly, being a retrospective study that utilizes the SEER database, it may be susceptible to inherent biases such as selection bias and limited generalizability to Asian patients due to the predominantly white population in the database. Furthermore, the lack of information on systemic treatment, specific chemotherapy regimens, radiation dose, endocrine therapy, and genetic markers may impact the accuracy of our models and necessitates further research. Secondly, our study only accounts for BM at the time of initial diagnosis, failing to account for the possibility of BM occurring later in the disease course. Lastly, our nomograms may be subject to overfit bias due to the exclusive use of the SEER dataset for both training and validation cohorts, highlighting the need for external validation with diverse clinicopathological data. To address these limitations, future studies should consider well-designed prospective analyses that incorporate more comprehensive data, including genetic markers, and construct predictive models that encompass both clinicopathological information and genetic markers.

## Conclusions

We’ve created a precise predictive model for prostate cancer patients with bone metastasis, using common clinical indicators to accurately address patient inquiries. Our model empowers clinicians and patients with clearer prognoses, enhancing treatment decision-making. We also developed prognostic tools and a risk system for prostate adenocarcinoma bone metastasis, laying the groundwork for personalized treatment. However, further research is needed to optimize treatment for these patients.

## Data Availability

This study used and/or analyzed data from the Surveillance, Epidemiology, and End Results (SEER) 18 Registries Data (https://seer.cancer.gov).

## Abbreviations

AUC: Area under the curve
CSS: Cancer-specific survival
CIF: Cumulative Incidence Function
DCA: Decision curve analysis
PC: Prostate cancer
PABM: Prostate adenocarcinoma with bone metastasis
PSA: prostate-specific antigen
IDI: Integrated differentiation improvement
NRI: Net reclassification improvement
OS: Overall survival
ROC: Receiver operating characteristic
SEER: Surveillance, Epidemiology, and End Results Program
